# Prevalence and antimicrobial resistance patterns of microbes isolated from individuals attending private diagnostic centre in Cape Coast Metropolis of Ghana

**DOI:** 10.1038/s41598-022-18595-w

**Published:** 2022-08-22

**Authors:** Alberta Serwah Anning, Emmanuel Baah, Suzzana Dickson Buabeng, Bernice Gloria Baiden, Benjamin Aboagye, Yeboah Kwaku Opoku, Leslie Larry Afutu, George Ghartey-Kwansah

**Affiliations:** 1 Department of Biomedical Sciences, College of Health and Allied Sciences, University of Cape Coast, Cape Coast, Ghana; 2 Department of Forensic Sciences, College of Agricultural and Natural Sciences, University of Cape Coast, Cape Coast, Ghana; 3 Department of Biology Education, University of Education, Winneba, Ghana; 4 Cocoa Clinic, Kejebril-Takoradi, Apowa Road, Ghana

**Keywords:** Biochemistry, Genetics, Microbiology

## Abstract

The evidence of rising numbers of multidrug-resistant organisms requires the implementation of effective stewardship programs. However, this should be informed by evidence-based knowledge of local antimicrobial resistance patterns. The current study aims to establish the prevalence of common pathogenic microbes including their antimicrobial susceptibility patterns and distribution in the Cape Coast Metropolis. This was a retrospective study where microbial culture and antimicrobial susceptibility records for 331 patients were reviewed from January to December 2019, at a private health centre. All data were analysed using Excel (Microsoft Office, USA), SPSS and GraphPad Prism 8 software programs. Among the samples tested, 125 (37.76%) were positive for microbes with high vaginal swab (HVS) samples recording the highest number of pathogens (44%), followed by urine (40%) and both pleural and semen samples having the least (0.3% each). Again, gram-negative isolates were more prevalent than the gram-positive isolates. The prevalence of antimicrobial resistance was very significant with isolates resistant to more than one antibiotic (P < 0.05). *Escherichia coli* showed the highest level of resistance, followed by *Citrobacter* spp. These were followed by *Klebsiella* spp.,* Staphylococcus* spp., Coliforms, *Pseudomonas* spp., Commensals and *Candida* spp. The high resistance pattern suggests an inevitable catastrophe requiring continuous monitoring and implementation of effective antibiotic stewardship.

## Introduction

Antimicrobial resistance (AMR) arises when microbes advance mechanisms that guard them from the effects of antimicrobials^1^. However, antibiotic resistance (AR) is when bacteria develop the ability to survive exposure to antibiotics^1^. Resistant microbes are difficult to treat, requiring higher doses, or alternative medications that may prove more toxic and expensive. Whereas microbes that are resistant to multiple antimicrobials are called multidrug-resistant (MDR), those that are known as extensively drug-resistant (XDR) or totally drug-resistant (TDR) are also called “superbugs”^2^. Resistance can occur naturally due to chance mutations. However, protracted use of antimicrobials encourages selection for mutations which can make antimicrobials ineffective. Furthermore, the lack of swift and proper identification of pathogens especially in patients with critical infection leads to broad-spectrum antibiotic overuse. Therefore, the prevention of antibiotic misuse can lead to a significant reduction in antibiotic resistance^3^. Narrow-spectrum antibiotics are favored over broad-spectrum antibiotics due to their effectiveness and accuracy in targeting specific organisms with less side effects^4^. For those who engage in self-medication, education about the detrimental effects of their actions is required. Health care providers can engage in antimicrobial stewardship to decrease the heavy load of antibiotic resistance^5^. Rising drug resistance is caused mainly by the use of antimicrobials in humans and other animals, and the spread of resistant strains between the two. Increasing resistance has also been associated with the discarding of inadequately treated wastes from the pharmaceutical industry, particularly in countries where majority of drugs are manufactured^6^. Antimicrobial resistance is increasing globally because of greater access to antibiotic drugs in developing countries^7^. A recent study estimates that 700,000 to several million deaths result per year and continues to pose a major public health threat worldwide due to bacterial resistance^8^. According to the world health organization (WHO) estimates, 350 million deaths could be caused by AMR by 2050^1^, thereby calling on the public for global collective action to address the threat that includes proposals for international treaties on antimicrobial resistance, as poorer countries with weaker healthcare systems are often more affected^3^.

Accurate information on the use of antibiotics is crucial to address the problem of antibiotic overuse and resistance^9^. Constant assessment of antibiotic use is necessary to preserve the efficacy of antibiotics and reduce harm to patients. The WHO recommends the surveillance of antibiotic use as a strategy for improving antibiotic use among patients and also for controlling antibiotic resistance^1^. Since health facilities are prone to nosocomial infections caused by hostile pathogens, the current study aimed to establish the prevalence of common pathogenic bacteria including their antimicrobial susceptibility patterns and distribution in the Cape Coast Metropolis through a 1-year retrospective study.

## Results

### Study population and demographics

In this study, patients were characterized into their sex and age groups respectively. Out of 331 patients recruited in the study, there were 105 males (31.7%) and 226 females (68.3%) represented in Table 1. On the tests conducted at the facility, urine analysis was observed to be the highest (51.1%), followed by high vaginal swab (HVS), (26.9) with pleural and semen analyses being the lowest (0.3% each).

**Table 1:** Number of tests conducted.

Test	Female	Male	Total number	Percentage (%)
Blood	8	4	12	3.6
Cervical	6	–	6	1.8
HVS	89	–	89	26.9
Pleural	1	–	1	0.3
Semen	–	1	1	0.3
Stool	9	8	17	5.1
Throat	–	1	1	0.3
Urethral	1	13	14	4.2
Urine	100	69	169	51.1
Wound	12	9	21	6.3
Total	226 (68.3%)	105 (31.7%)	331	100

### Prevalence of microbial isolates

**Figure 1:**
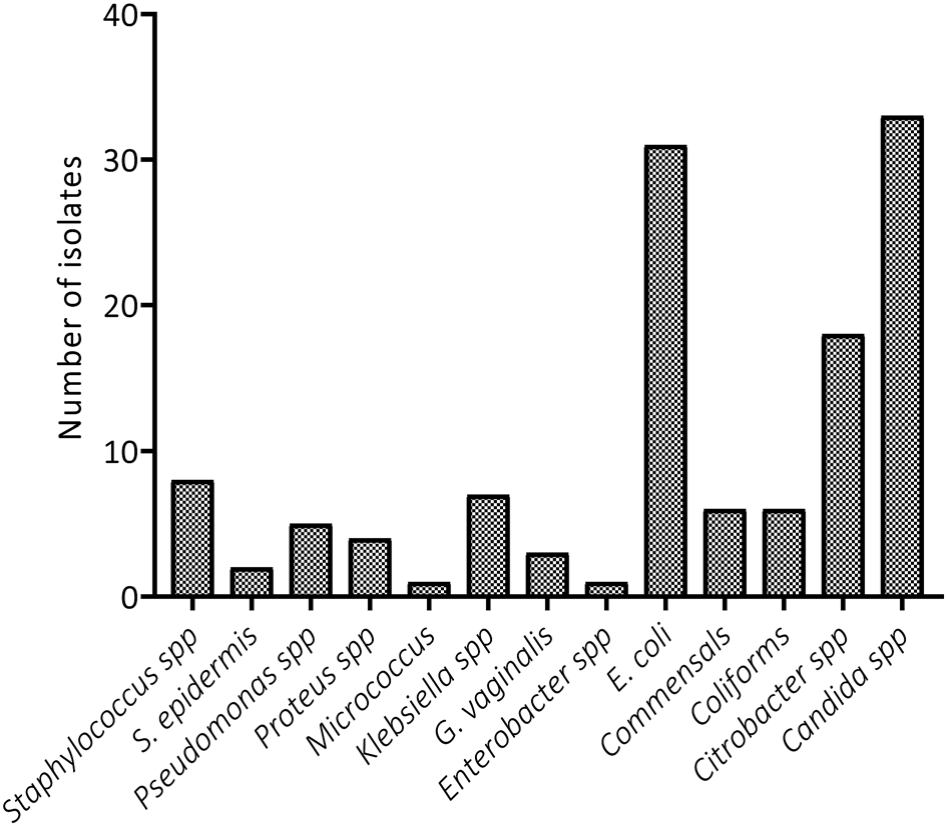
Type and frequency of microorganisms isolated from the patient visiting the health facility.

**Figure 2:**
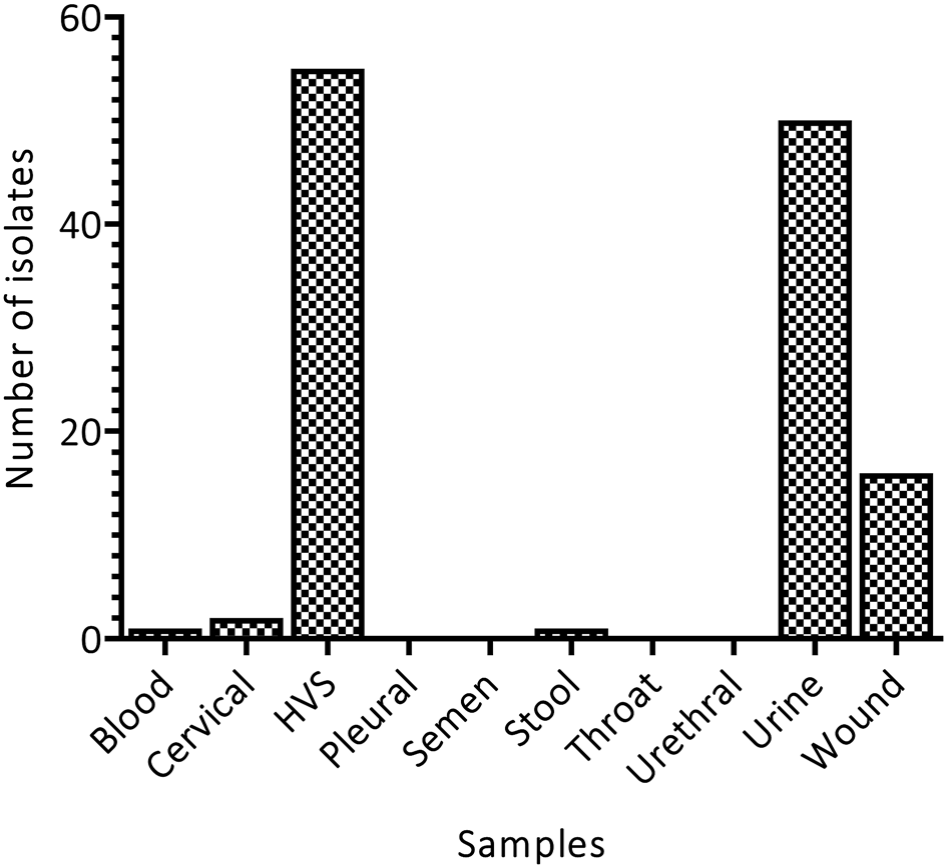
Distribution of different clinical isolates among the various samples.

The overall number of individual isolates and the prevalence of pathogens during the study period of 1 year (P < 0.05) were established and compared using one-way ANOVA analysis. *Candida* spp. were the most abundant, followed by *E. coli*, with the least being both *Enterobacter* and *Micrococcus* (Fig. 1 and Table 2). In terms of samples, HVS recorded the highest prevalence of bacteria followed by urine and wound. However, no bacteria were detected in the urethra, throat, semen and pleural (Fig. 2, One-way ANOVA, P < 0.05)*.*

**Table 2:** Prevalence of Microorganisms in samples.

Microbe	Number of samples of tests within which microbes were identified									
	Blood	Cervical	HVS	Pleural	Semen	Stool	Throat	Urethra	Urine	Wound
*Candida* spp*.*	*–*	1	24	–	–	–	–	–	8	–
*Citrobacter* spp.	*–*	–	7	–	–	–	–	–	10	1
*Coliforms*	*–*	–	–	–	–	–	–	–	–	6
*Commensals*	*–*	–	6	–	–	–	–	–	–	–
*E. coli*	*–*	1	7	–	–	–	–	–	23	–
*Enterobacter* spp.	*–*	–	–	–	–	–	–	–	1	–
*G. vaginalis*	*–*	–	3	–	–	–	–	–	–	–
*Klebsiella* spp.	*–*	–	–	–	–	–	–	–	6	1
*Micrococcus*	*–*	–	–	–	–	–	–	–	1	–
*Proteus* spp.	*–*	–	1	–	–	1	–	–	1	1
*Pseudomonas* spp.	*–*	–	–	–	–	–	–	–	–	5
*S. epidermis*	1	–	1	–	–	–	–	–	–	–
*Staphylococcus* spp.	*–*	–	6	–	–	–	–	–	–	2

### Distribution of microbes according to demographics

**Figure 3:**
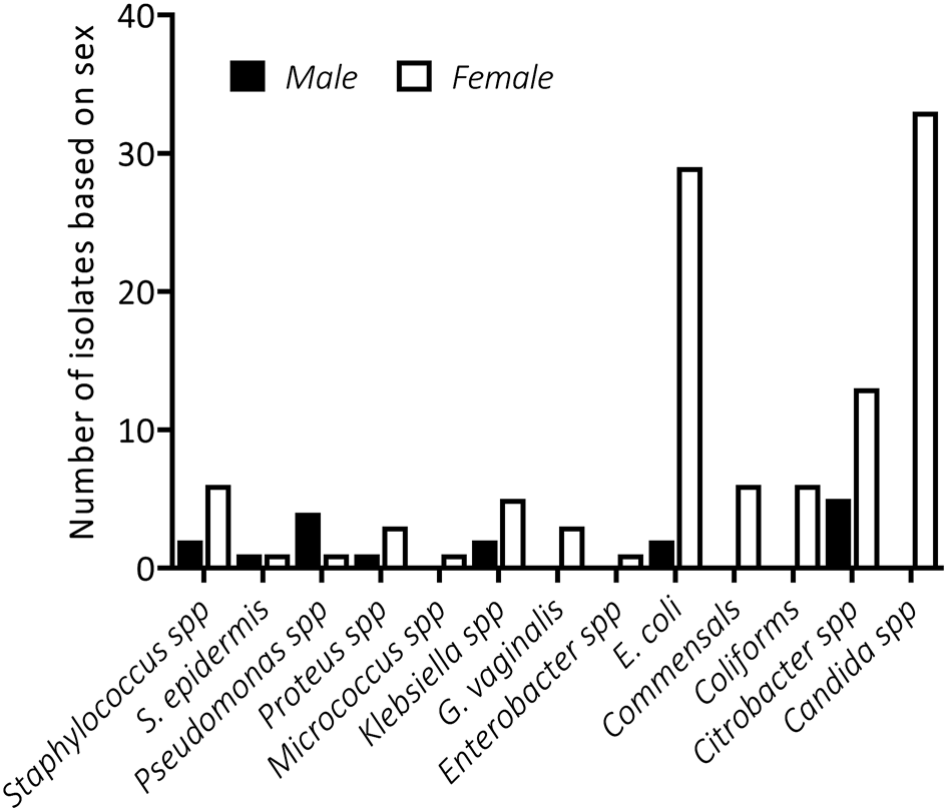
Gender-specific distribution of microbes.

To identify the group that was more susceptible to bacterial infections, isolates were distributed according to age and sex of which *Citrobacter* spp., *E. coli*, *Klebsiella* spp., *Proteus* spp., among others were identified in both sexes. On the other hand, however, *Enterobacter* spp. was seen only in males whereas *Candida* spp., *G. vaginalis*, and *Micococcus* were identified in females only. *Candida* spp. and *E. coli* were the most common isolates (Fig. 3, Two-way ANOVA, P < 0.05). Other Coliform and Commensal bacteria were present but few.

Participants aged 26–40 years recorded the highest number of microbes followed by age groups 16 to 25 years and 60 years and above (Table 3). In addition, the distribution of the microbes was not in an age-specific manner. As indicated earlier, *Candida* spp. and *E. coli* were observed to be the bacteria with the highest prevalence. *Candida* spp. was more in the 16–25 and 26–40 age groups, whereas *E. coli* was prominent in the 26–40 and ≥ 60 years age groups (Table 4). Again, high prevalence of *Candida* spp. and *E. coli* infections in the age group 26–40 years were observed.

**Table 3:** Prevalence of microorganisms among age groups.

Age	Number of samples		
	Total	Microbe absent	Microbe present
1–5	8	7	1
6–15	9	6	3
16–25	51	27	34
26–40	145	96	49
41–60	41	31	10
Above 60	67	35	32

**Table 4:** Individual bacteria identified in samples per age groups.

Microbe	Age groups					
	1–5	6–15	16–25	26–40	41–60	> 60
*Candida* spp.	0	0	12	19	1	1
*Citrobacter* spp.	0	2	4	3	2	7
*Coliforms*	0	0	0	2	3	1
*Commensals*	0	0	1	3	1	1
*E. coli*	0	2	3	14	2	10
*Enterobacter* spp.	0	0	0	0	1	0
*G. vaginalis*	0	0	1	1	0	1
*Klebsiella* spp.	0	0	1	3	0	3
*Micrococcus*	0	0	1	0	0	0
*Proteus* spp.	0	0	0	1	0	3
*Pseudomonas* spp.	0	0	0	2	1	2
*S. epidermis*	1	0	0	1	0	0
*Staphylococcus*	0	0	2	3	0	3

### Antimicrobial susceptibility profiles of microbes

**Figure 4:**
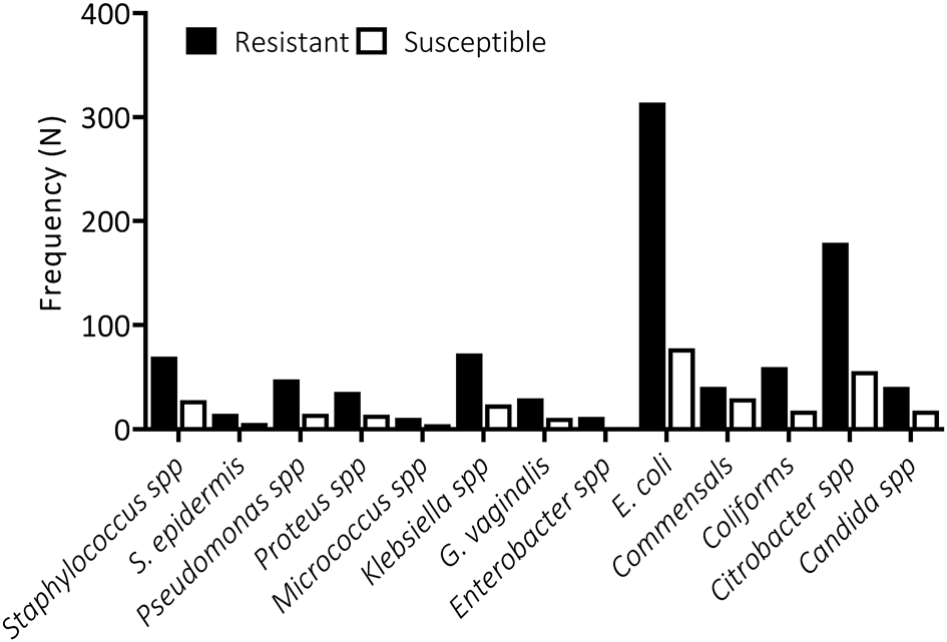
Prevalence of antimicrobial resistance among the specific microbe isolates.

All isolates were tested for their susceptibility or resistance to the most commonly used antimicrobials at the diagnostic centre during the one-year period. All bacteria isolates showed resistance to at least two antibiotics (Fig. 4, Supplementary Table S1a,b, P < 0.05, Two-way ANOVA), hence, a disturbing level of antimicrobial resistance was registered in the study. *E. coli* showed the highest resistance level among all the pathogens, followed by *Citrobacter* spp., *Klebsiella* spp., *Staphylococcus* spp., *Coliforms*, *Pseudomonas* spp., Commensals and then lastly *Candida* spp. Interestingly, *Enterobacter* spp. was not susceptible to any of the antibiotics. The microbes were remarkably resistant to Cloxacillin with significantly low susceptibility. Interestingly, microbes showed significantly high susceptibility and relatively low resistance to Amikacin. It is worthy of note that only one *Enterobacter* was resistant to 12 out of the 14 antibiotics tested (Supplementary Table S1b). The prevalence of antibiotic resistance was very significant among both gram-negative and gram-positive organisms. This high resistance pattern foreshadows an inevitable catastrophe that requires continuous monitoring and implementation of effective antibiotic policies.

## Discussion

Understanding the distribution of microbial pathogens and their associated infections is required to control infectious diseases and monitor antimicrobial resistance. The current study aimed at establishing the prevalence of common pathogenic microorganisms including their antimicrobial susceptibility patterns and distribution according to specimens, age groups and sex at a private diagnostic Centre in the Cape Coast Metropolis.

The excessive use of antibiotics among other factors has led to extensive antimicrobial resistance. If this trend continues unabated, then all other antibiotic options will be exhausted making the treatment of associated infections extremely difficult. Hence, the WHO identified it as an international health problem of prime concern^10–12^. To control this rising predicament, all-inclusive antibiotic and other relevant stewardship especially in poor countries are essential. However, enough data concerning antimicrobial resistance are inaccessible to exactly measure the degree of the problem. The few available studies regarding results on microbiological samples suggest that there are hotbeds of emerging high-level resistance^10^.

In this study, gram-negative bacteria were more prevalent than gram-positive isolates, similar to reports by Newman and colleagues, and Fahim^10,13^. Most of the isolates were recovered from HVS samples representing 44%, followed by urine samples which recorded 40% of the total samples that contained pathogens unlike the results of Fahim who reported higher recovery from blood, followed by urine specimens^10^. Gram-negative bacteria cause various infections including pneumonia, bloodstream infections, wound or surgical site infections, and meningitis among others. Gram-negative bacteria are resistant to multiple drugs with suggested development of resistance to most of the available antibiotics. This observation can be attributed to their in-built abilities to find alternative ways to develop resistant and thus, causing significant morbidity and mortality worldwide^14^.

The high prevalence of microbial isolates reported in this study highlights the need for effective monitoring and surveillance of microbial infections in resource-limited health care facilities^15^.

Among all microorganisms isolated, *Candida* spp. was seen to be most abundant followed by *E. coli*, with the least being both *Enterobacter* and *Micrococcus*. Among the gram-negatives, *E. coli* represented the most isolated pathogen while *Enterobacter* spp. was the least whereas in the gram-positive isolates, *Candida* spp. (fungus) represented the most isolated pathogen whiles *Micrococcus* spp. was the least. In Nigeria, Osifo and Aghahowa reported that *E. coli* and *Klebsiella pneumoniae* were the most frequently isolated pathogens^16^. Invasive *Candida* infections are often associated with high rates of morbidity and mortality^17^, therefore, the high levels observed in the current study is a cause for concern. While *E. coli* is a normal resident of the healthy gut, it is also an important and widespread pathogen which has been associated with human infections including diarrhoea, urinary tract infections and meningitis.

To identify the most vulnerable group from bacterial infections, isolates were distributed according to sex and age groups of the patients. There were more pathogens in females (226, representing 68.3%) than in males (105 representing 31.7%) and in some cases found in only females (*Candida* spp. and *G. vaginalis* spp.) with the exception of *Pseudomonas* spp., which was higher in males (4 representing 80%) than females (1 representing 20%). This observation deviates from what has been reported elsewhere, where the distribution in males and females were virtually the same, thus, 51% and 49% respectively^15^ but similar to that of Mapanguy and colleagues who reported a significantly higher prevalence among females (61%) than males (39%)^18^. Also, a significant number of bacteria were isolated from the age group 26–40 years, followed by 60 and above with age group 1–5 years recording the least. The study recruited more adults than children hence the observation that more isolates were obtained from adults corresponds with the high number of adult clients recorded.

We report a high prevalence of microorganisms with variable susceptibility patterns to key antimicrobials. All microorganisms isolated showed resistance to more than one antimicrobial agent. Cotrimoxazole, Erythromycin, Vancomycin, Chloramphenicol and Cefuroxime were among the top five antimicrobials with a high prevalence of resistance. However, Amikacin, Gentamicin and Nitrofurantoin were the three most effective antibiotics. This is similar to an earlier report where amikacin was among the group with lowest resistance^13^. Furthermore, Fahim also reported in Egypt that gram-negative isolates exhibited high resistance to almost all the classes of antibiotic in use with the least frequency recorded against nitrofurantoin, amikacin, followed by imipenem and meropenem^10^.

*Escherichia coli* was the pathogen with the highest resistance and the highest resistance was toward cefuroxime, chloramphenicol, meropenem, vancomycin and erythromycin. The next resistant microbe was *Citrobacter* spp., which was highly resistant only to chloramphenicol. Conversely, *E. coli* and *Citrobacter* spp. were highly susceptible to amikacin. This is similar to a study in Congo, where *E. coli* was the highly resistant ceftazidime, followed by amoxicillin, piperacillin-tazobactam, ofloxacin, and azithromycin^18^. Also, a previous report among healthy individuals in an Indian population showed similar patterns of resistance^19^. Interestingly, a high prevalence of resistance to ceftazidime was reported in a study in Uganda^20^ and amoxicillin in Nigeria^21^.

Apart from *E. coli* and *Citrobacter* spp.,* Klebsiella* spp., *Staphylococcus* spp., other coliforms, *Pseudomonas* spp., *Candida* spp. and other commensals were among the most resistant microbes isolated. Other studies have reported similar results where the most prevalent organisms in the collection included *E. coli*, *S. aureus*, *Klebsiella* spp., *Pseudomonas aeruginosa*,* Citrobacter* spp. and *Enterobacter* spp.^13,22^.

Factors that may have contributed to the emergence and prevalence of resistance, includes uncontrolled use of these drugs, non-compliance with treatment and geographical location/unsanitary environment. Another significant factor for increased resistance to antibiotics is the use of substandard and counterfeit drugs, and the unauthorized sale of antibiotics without prescription^18,23,24^. Interestingly, *Enterobacter* spp. was not susceptible to any of the antibiotics whereas the majority of the microbes were remarkably resistant to Cloxacillin with lower susceptibility levels. In contrast, Amikacin showed high activity towards these microorganisms. This means that amikacin is the antibiotic effective against the greatest number of microorganisms characterized in this study. Correspondingly in another study, a lower percentage of resistance was observed for ceftriaxone, ciprofloxacin, and amikacin^13^.

It is significant to note that this study provides a general overview of the current shocking situation in the area under study. This implies that urgent action needs to be taken to halt this catastrophic menace by starting an effective action plan for its containment.

## Conclusion

Gram-negative isolates were the most common bacteria isolated from patients attending this referral laboratory service compared to the gram-positive isolates. Of these, *E. coli* represented the most isolated pathogen while *Enterobacter* spp. However, in the gram-positive isolates, *Candida* spp. represented the most isolated pathogen whiles *Micrococcus* spp. was the least. The prevalence of antimicrobial resistance was very significant among the isolated pathogens. The highest resistance was found in *Escherichia coli* and the highest resistance was toward cefuroxime, chloramphenicol, meropenem, vancomycin and erythromycin. The increased antimicrobial resistance reported in the study could be due to the unreasonable use of antibiotics by the populace. Nonetheless, to fight against antimicrobial resistance, a localized epidemiological surveillance program is required to help establish evidence-based guidelines for the treatment and management of microbial infections. The observed high resistance pattern also requires continuous monitoring and implementation of effective antibiotic stewardship.

## Method

### Study area and design

The study was conducted at a Private Diagnostic Centre in the Cape Coast Metropolis, Central Region, Ghana. The facility serves as a referral diagnostic centre in the Central Region of Ghana. This study is a retrospective analysis of routine recovered bacterial isolates subjected against a panel of antibiotics for susceptibility testing^25^. The study spun from January to December 2019.

### Data extraction

A retrospective audit of records for three hundred and thirty-one (331) participants’ bacterial culture and susceptibility testing results from the month of January 2019 to December 2019 from a private diagnostic centre was conducted. This Private diagnostic centre is a referral unit where laboratory tests from various hospitals within the Cape Coast Metropolis are sent. By using a Convenient sampling technique, records of specimens such as urine, stool, blood, and various body sites (cervical, wound, etc) were all included.

### Processing and identification of isolates

Sample processing and identification of the isolates were performed per the standard operating procedures (SOPs) of the laboratory. The samples were cultured on the routinely used microbiological media and incubated for 24 h at 37 °C^10^. If no growth, the plates were incubated for a total of 48 h. The identification of the isolates was done according to colony morphology, gram stain, and standard confirmatory biochemical tests. Gram-positive bacteria were identified by testing the hemolytic activity on blood agar and further identification using different biochemical tests such as catalase reaction, slide and tube coagulase tests, culture on DNase agar, bile esculin, in addition to different differentiating antibiotic discs such as optochin and bacitracin. For gram-negative bacteria, identification was conducted by biochemical tests such as oxidase, triple sugar iron, motility indole ornithine, citrate, lysine iron arginine, and urease tests^10^.

### Antimicrobial susceptibility

Antimicrobial susceptibility tests of the isolates were performed using the Kirby–Bauer disk diffusion method and interpreted according to the Clinical Laboratory Standards Institute (CLSI) guidelines^26^. Briefly, a standardized is swabbed onto the surface of MH agar. Since reproducibility depends on the log growth phase of organisms, fresh subcultures are used. Filter paper disks impregnated with a standardized concentration of an antimicrobial agent were placed on the surface, and the size of the zone of inhibition around the disk is measured after overnight incubation. Specific incubation time ranges were outlined in the Clinical and Laboratory Standards Institute [CLSI] documents^27^.

### Data analysis

All data were analysed using statistical package for social sciences (SPSS) computer software (Version 25) and GraphPad Prism 8 software (San Diego, CA, USA). Graphs were used to show the prevalence and distribution of the isolated bacteria. In addition, a frequency table expressed in percentages and absolute numbers were used to display the susceptibility patterns of the commonly isolated bacteria against the commonly used antibiotics. A statistically significant difference was considered at a P-value of ≤ 0.05.

### Ethical approval

Ethical approval was obtained from the Cape Coast Teaching Hospital Ethical Review Committee (CCTHERC) with reference number CCTHERC/EC/2021/013 for data acquisition. All methods were carried out in accordance with relevant guidelines and regulations. Informed consent was obtained from all subjects and/or their legal guardian(s).

## Supplementary Information

Supplementary Information.

## Data Availability

All data generated or analysed during this study are included in this published article [and its Supplementary Information files].

## Abbreviations

BSI: Bloodstream infection
FBC: Full blood count
HIV: Human immunodeficiency virus
HVS: High vaginal swab
IBI: Invasive bacterial infection
NTS: Non-typhoidal salmonella
UTI: Urinary tract infection
VDRL: Venereal Disease Research Laboratory
WBC: White blood count
WHO: World Health Organization
