# Supplementary material for: Prevalence and antimicrobial resistance patterns of microbes isolated from individuals attending private diagnostic centre in Cape Coast Metropolis of Ghana
Source: Sci Rep. 2022 Aug 22;12:14282. doi: 10.1038/s41598-022-18595-w (PMC9395395; doi:10.1038/s41598-022-18595-w)
Supplement: Supplementary file 1 — Supplementary Information. [file 41598_2022_18595_MOESM1_ESM.docx]

**Supplementary Data**

**Table S1a:** **Antimicrobial susceptibility patterns of major pathogens against commonly used antimicrobials**

| ANTIBIOTICS | | | | | | | | | | |  |
| --- | --- | --- | --- | --- | --- | --- | --- | --- | --- | --- | --- |
|  | Ceftriaxone | Meropenem | Nitrofurantoin | Cefotaxime | Chloramphenicol | Tetracycline | Vancomycin | Norfloxacin | Ciprofloxacin | Erythromycin |  |
|  |  |  |  |  |  |  |  |  |  |  |  |
| MICROBES | *S (%)*  *R (%)* | *S (%)*  *R (%)* | *S (%)*  *R (%)* | *S (%)*  *R (%)* | *S (%)*  *R (%)* | *S (%)*  *R (%)* | *S (%)*  *R (%)* | *S (%)*  *R (%)* | *S (%)*  *R (%)* | *S (%)*  *R (%)* |  |
| *E. coli* | 9 (30)  21(70) | 2 (7)  28 (93) | 0 (0)  1 (100) | 0 (0)  30 (100) | 1 (3)  30 (97) | 2 (9)  21 (91) | 2 (7)  28 (93) | 6 (26)  17 (74) | 10 (33)  20 (67) | 0 (0)  27 (100) |  |
| *Candida spp.* | 3 (75)  1(25) | 1 (25)  3 (75) | 5 (50)  5 (50) | 1 (25  3 (75) | 0 (0)  4 (100) | NA | 1 (33)  2 (67) | 0 (0)  1 (100) | 0 (0)  4 (100) | 0 (0)  4 (100) |  |
| *Enterobacter spp.* | 0 (0) 1(100) | 0 (0)  1 (100) | NA | 0 (0)  1 (100) | 0 (0)  1 (100) | 0 (0)  1 (100) | 0 (0)  1 (100) | 0 (0)  1 (100) | 0 (0)  1 (100) | 0 (0)  1 (100) |  |
| *Citrobacter spp.* | 5 (29)  12(71) | 1 (6)  16 (94) | NA | 5 (28)  13 (72) | 1 (6)  17 (94) | 0 (0)  11 (100) | 1 (6)  17 (94) | 1 (9)  10 (91) | 8 (44)  10 (56) | 1 (6)  16 (94) |  |
| *Klebsiella spp.* | 2 (29) 5 (71) | 1 (17)  5 (83) | 0 (0)  1 (100) | 0 (0)  7 (100) | 0 (0)  7 (100) | 0 (0)  6 (100) | 0 (0)  7 (100) | 1 (17)  5 (83) | 4(57)  3 (43) | 0 (0)  7 (100) |  |
| *Coliforms* | 0 (0)  6(100) | NA | NA | 1 (17)  5 (83) | 1 (17)  5 (83) | 1 (17)  5 (83) | 0 (0)  6 (100) | NA | 3 (50)  3 (50) | 0 (0)  6 (100) |  |
| *Proteus spp.* | 2 (50)  2 (50) | 0 (0)  2 (100) | NA | 1 (25)  3 (75) | 0 (0)  4 (100) | 0 (0)  2 (100) | 1 (33)  2 (67) | 1 (50)  1 (50) | 2 (50)  2 (50) | 0 (0)  3 (100) |  |
| *Staphylococcus spp.* | 0 (0)  8(100) | 3 (50)  3 (50) | NA | 1 (12.5)  7 (87.5) | 1 (12.5)  7 (87.5) | 0 (0)  2 (100) | 2 (25)  6 (75) | NA | 6 (75)  2 (25) | 3 (37)  5 (63) |  |
| *Pseudomonas spp.* | 0 (0) 5(100) | NA | NA | 0 (0)  5 (100) | 0 (0)  5 (100) | 0 (0)  5 (100) | 0 (0)  5 (100) | NA | 5 (100)  0 (0) | 0(0)  3 (100) |  |
| *Commensals* | 0 (0)  4(100) | 1 (25)  3 (75) | 16 (70)  7 (30) | 0 (0)  4 (100) | 1 (25)  3 (75) | NA | 1 (25)  3 (75) | NA | 4 (100)  0 (0) | 1 (25)  3 (75) |  |
| *G. vaginalis* | 0 (0)  (100) | 0 (0)  3 (100) | 2 (33)  4 (67) | 1 (33)  2 (67) | 1 (33)  2 (67) | NA | 0 (0)  3 (100) | NA | 1 (33)  2 (67) | 0 (0)  3 (100) |  |
| *Micrococcus spp.* | 0(0)  1(100) | 0 (0)  1 (100) | 2(100)  0 (0) | 0 (0)  1 (100) | 0 (0)  1 (100) | 0 (0)  1 (100) | 1 (100)  0 (0) | 0 (0)  1 (100) | 1 (100)  0 (0) | 0 (0)  1 (100) |  |
| *S. epidermis* | NA | 0 (0)  1 (100) | NA | 0 (0)  2 (100) | 0 (0)  2 (100) | 1 (100)  0 (0) | 0 (0)  2 (100) | NA | 1 (50)  1 (50) | 1 (50)  1 (50) |  |
| *Total* | 9 (12)  69(88) | 9 (12)  66 (88) | 25 (58)  18 (42) | 10 (11)  83 (89) | 6 (6)  89 (94) | 4 (7)  54 (93) | 9 (10)  82 (90) | 9 (20)  36 (80) | 45 (48)  48 (52) | 6 (7)  80 (93) |  |

**Key**: **NA** Not Applicable, **S** Susceptible, **R** Resistance

**Table S1b:** **Antimicrobial susceptibility patterns of major pathogens against commonly used antimicrobials**

|  |  |  |  | ANTIBIOTICS |  |  |  |  |
| --- | --- | --- | --- | --- | --- | --- | --- | --- |
|  | Levofloxacin | Amikacin | Piperacilin | Co-Trimoxazole | Gentamicin | Ampicillin | Cloxacillin | Penicillin |
| MICROBES | S (%)  R (%) | S (%) R (%) | S (%)  R (%) | S (%)  R (%) | S (%)  R (%) | S (%)  R (%) | S (%)  R (%) | S (%)  R (%) |
| *E. coli* | 5 (21)  19 (79) | 24(86) 4 (14) | 5 (22) 18 (78) | 4 (14)  25 (86) | 14 (48) 15 (52) | 3 (37) 5 (63) | NA | 2 (29) 5 (71) |
| *Candida spp.* | 0 (0)  1 (100) | 4 (100) 0 (0) | NA | 0 (0)  4 (100) | 1 (25) 3 (75) | 2 (50) 2 (50) | NA | 0 (0) 4 (100) |
| *Enterobacter spp.* | NA  1 (100) | 1 (100) 0 (0) | 0 (0) 1 (100) | 0 (0)  1 (100) | 1 (100) 0 (0) | NA | NA | NA |
| *Citrobacter spp.* | 2 (18)  9 (82) | 17 (100) 0 (0) | 0 (0) 11 (100) | 4 (22)  14 (78) | 7 (39) 11 (61) | 2 (33) 4 (67) | 0 (0)  1 (100) | 1 (12.5) 7 (87.5) |
| *Klebsiella spp.* | 3 (50)  3 (50) | 6 (86) 1 (14) | 0 (0) 6 (100) | 0 (0)  7 (100) | 6 (86) 1 (14) | 0 (0) 1 (100) | 0 (0) 1 (100) | NA |
| *Coliforms* | NA | 6 (100) 0 (0) | NA | 1 (17)  5 (83) | 4 (67) 2 (33) | 0 (0) 6 (100) | 0 (0)  6 (100) | 1 (17) 5 (83) |
| *Proteus spp.* | 0(0)  2 (100) | 4 (100) 0 (0) | 0 (0) 1 (100) | 1(25)  3 (75) | 1 (25) 3 (75) | 1 (33) 2 (67) | 0 (0)  1 (100) | 0 (0) 3 (100) |
| *Staphylococcus spp.* | NA | 0 (0) 8 (100) | NA | 2 (25)  6 (75) | 7 (87)  1 (13) | 0 (0) 8 (100) | 1 (50) 1 (50) | 2 (25) 6 (75) |
| *Pseudomonas spp.* | 0(0)  1(100) | 3 (75) 1 (25) | 1 (100) 0 (0) | 1 (25)  3 (75) | 2(40) 3 (60) | 1 (20) 4 (80) | 0 (0) 5 (100) | 2 (40) 3 (60) |
| *Commensals* | NA | 0 (0) 4 (100) | NA | 0 (0)  4 (100) | 4 (100) 0 (0) | 0 (0)  4 (100) | NA | 2 (50) 2 (50) |
| *G. vaginalis* | NA | 1(33) 2 (67) | NA | 0 (0)  3 (100) | 3 (100) 0 (0) | 0 (0)  2 (100) | NA | 2 (67) 1 (33) |
| *Micrococcus spp.* | (0)  1 (100) | 0 (0) 1 (100) | 0 (0) 1 (100) | 0 (0)  1 (100) | 1 (100) 0 (0) | NA | NA | NA |
| *S. epidermis* | 0 (0)  1 (100) | 1 (50) 1 (50) | NA | 0 (0)  1 (100) | 2 (100) 0 (0) | 0 (0) 1 (100) | NA | 0 (0) 2 (100) |
| Total | 10 (21)  38 (79) | 67 (75) 22 (25) | 6 (14) 38 (86) | 13 (14)  77 (86) | 53 (58) 39 (42) | 9 (19) 39 (81) | 1 (6) 15 (93) | 12 (24) 38 (76) |

**Key**: **NA** Not Applicable, **S** Susceptible, **R** Resistance
